# Mothers’ experiences in the Nurse-Family Partnership program: a qualitative case study

**DOI:** 10.1186/1472-6955-11-15

**Published:** 2012-09-06

**Authors:** Christine Kurtz Landy, Susan M Jack, Olive Wahoush, Debbie Sheehan, Harriet L MacMillan

**Affiliations:** 1 School of Nursing, York University, 4700 Keele Street, HNES, Toronto, ON M3J 1P3, Canada; 2 School of Nursing, McMaster University, 1280 Main Street West, Hamilton, ON L8S 4K1, Canada; 3 Faculty of Health Sciences, Simon Fraser University, 515 West Hastings Street, Vancouver, BC, V6B 5K3, Canada; 4 Departments of Psychiatry and Behavioural Neurosciences and Pediatrics, Offord Centre for Child Studies, McMaster University, Patterson Building, 1200 Main Street West, Hamilton, ON L8N 3Z5, Canada

**Keywords:** Nurse home visiting, Low income, Young mothers, Mother’s experiences

## Abstract

**Background:**

Few studies have explored the experiences of low income mothers participating in nurse home visiting programs. Our study explores and describes mothers' experiences participating in the Nurse-Family Partnership (NFP) Program, an intensive home visiting program with demonstrated effectiveness, from the time of program entry before 29 weeks gestation until their infant's first birthday.

**Methods:**

A qualitative case study approach was implemented. A purposeful sample of 18 low income, young first time mothers participating in a pilot study of the NFP program in Hamilton, Ontario, Canada partook in one to two face to face in-depth interviews exploring their experiences in the program. All interviews were digitally recorded and transcribed verbatim. Conventional content analysis procedures were used to analyze all interviews. Data collection and initial analysis were implemented concurrently.

**Results:**

The mothers participating in the NFP program were very positive about their experiences in the program. Three overarching themes emerged from the data: 1. Getting into the NFP program; 2. The NFP nurse is an expert, but also like a friend providing support; and 3. Participating in the NFP program is making me a better parent.

**Conclusions:**

Our findings provide vital information to home visiting nurses and to planners of home visiting programs about mothers' perspectives on what is important to them in their relationships with their nurses, how nurses and women are able to develop positive therapeutic relationships, and how nurses respond to mothers' unique life situations while home visiting within the NFP Program. In addition our findings offer insights into why and under what circumstances low income mothers will engage in nurse home visiting and how they expect to benefit from their participation.

## Background

The Nurse-Family Partnership (NFP) is a targeted home visiting program provided by nurses to young, low-income, first-time mothers during pregnancy and through the first two years of the child’s life. The effectiveness of the NFP program has been rigorously evaluated in three US randomized controlled trials [1-4]. The goals of the NFP program include improving: (a) pregnancy outcomes, (b) child health and development through more responsible and competent parenting, and (c) families’ economic self-sufficiency by helping parents to plan for their future, continue their education or find work and plan subsequent pregnancies. Through these three U.S. trials, Olds and colleagues have repeatedly demonstrated that the NFP is effective and consistent findings include: 1. reductions in childhood injuries; 2. improved school readiness; 3. reductions in the number and increased spacing of subsequent pregnancies; 4. reductions in welfare dependence; and 5. increased maternal employment [1,2,5-7]. A longer-term study of the cohort of young adults whose mothers enrolled in the original Elmira, NY trial revealed a trend that teenage children of NFP participants were less involved in substance abuse and crime than teens whose mothers did not receive the intervention [8,9]. Eckenrode and colleagues reported that 19 year-old female youth whose mothers participated in the NFP program were less likely to be involved in crime, had fewer children and used less Medicaid [10]. In addition Kitzman and colleagues found that the positive effects of the NFP program on maternal life course endured three years after mothers completed the NFP intervention [11].

The NFP program follows set guidelines and commences with visits beginning before the 29^th^ week of pregnancy and extending to the child’s second birthday. NFP nurses visit biweekly except during the first month of the intervention and the first postpartum month, when nurses visit weekly. The nurses focus on six domains: personal health, environmental health, friends and family, the maternal role, use of health care and human services, and maternal life course development (which encompasses planning for future pregnancies, education, and employment). During pregnancy, the focus is on fetal growth; attachment; changes in the mother’s body and life; changes in relationships with her partner, family, and friends; and questions about her labor and delivery and how to integrate motherhood into responsibilities with school and work. After the birth, the focus broadens to encompass infant growth and development, educational play, bonding and communicating with her child, and the mother’s life-course planning. The mother’s participation in the program is voluntary.

Researchers studying the effectiveness of home visiting programs in general have hypothesized that positive maternal and child outcomes are related to the development of trusting relationships between the professional and the mother [1,12-15]. The success of the NFP has been attributed to the nurses’ development of therapeutic relationships with their clients [1]. Wiggins and colleagues suggest that it is the mix of well trained nurses, their strengths-based approach to families and the content and length of the program that sets the NFP apart from other home visiting programs [16], many of which have not been found to be effective [17,18]. Good therapeutic relationships between home visiting nurses and mothers require nurses to help mothers become empowered active participants to increase control over their own health [19,20]. In so doing, nurses must be non-judgmental in order to create a safe environment for the trusting relationship to develop [19]. However, Smithbattle points out that little is known about how nurses and women are able to cultivate these positive therapeutic relationships, and how nurses respond to clients’ unique life situations while following a set curriculum during home visits [13].

Few studies have examined mothers’ experiences and perceptions of home visiting by nurses. Jack and colleagues examined how socially disadvantaged mothers engage with public health nurses and home visitors [12]. They reported that women experienced vulnerability and powerlessness, and had to engage in social processes of overcoming fear, building trust and seeking mutuality in order to limit feelings of vulnerability. They found that provider actions, maternal characteristics, and past experiences influenced the speed with which mothers moved through phases in order to develop a connected relationship [11,21]. DeMay in her analysis of 62 essays written by mothers about their experiences receiving nurse home visits reported that the mothers emphasized the importance of nurse qualities, obtaining knowledge about pregnancy and child development and “feeling respected and not feeling the nurse was telling them what to do” (p.234) [21]. Other studies report that some adolescent mothers feel stigmatized by public health nurses during home visits because they are teen mothers [22].

Although several studies have examined and demonstrated the effectiveness of the NFP program, no published research was found that specifically examined the experiences of mothers participating in this nurse home visiting program. Knowledge of women’s experiences within and perceptions of the NFP program is important as it will provide insights into what women like and value about the program and factors that influence their continued participation in the program. In addition, the findings may provide some insight into the mechanisms, including how the relationship between the mothers and their NFP nurses improves maternal, child and family outcomes. The aim of this study was to explore and describe the experiences of mothers participating in the NFP program from the time of program entry before 29 weeks gestation until their infant’s first birthday. The results presented in this paper are part of a larger mixed methods study examining the feasibility and acceptability of the NFP in a Canadian setting.

The Prevention Research Centre (University of Colorado) Nurse Family Partnership International Program identified the McMaster University-Hamilton Public Health Services collaboration as the team to lead all future evaluations of the NFP in Canada. To retain fidelity to the program model evaluated in the original trials, international sites are required to complete a four-step process prior to full implementation of the program: 1) adapt the NFP curriculum to meet local standards and needs; 2) complete a small scale pilot study to assess for acceptability and feasibility; 3) conduct an RCT to evaluate the effectiveness of the program in the new context; and 4) expand implementation of the program. In 2008, a feasibility study was initiated in Hamilton, Ontario to evaluate procedures for recruitment, retention and collection of clinical data. Concurrently, a qualitative acceptability study was conducted to explore NFP clients’ and their families’, public health nurses’ (PHNs), and stakeholders’ perceptions of and experiences with this specific home visitation intervention.

Six PHNs and one nurse manager completed the intensive nurse and nurse manager NFP training. In order to ensure fidelity to the NFP program, all NFP nurses and their nurse supervisors receive training from the Nursing Practice Team at Nurse-Family Partnership National Service Office in Denver, Colorado, USA. The comprehensive training includes a theoretical review of the NFP program elements, an overview of NFP program elements and application of the curriculum, and advanced skill development in nursing assessment and intervention. Prior to implementing the program in Hamilton, the PHNs had an opportunity to job-shadow a team of NFP nurse home visitors located in Pennsylvania.

## Methods

In 2008, the pilot study to assess feasibility and acceptability was initiated in Hamilton, Ontario, Canada. Hamilton has approximately 6,000 births per year [23]. Twenty percent of Hamilton’s citizens live at or below the poverty line [24]. In 2007, 547 first-time mothers 21 years of age or younger gave birth in Hamilton [25].

A total of 108 pregnant women were recruited into the pilot study to receive the full NFP intervention until their child’s second birthday. The women were recruited through prenatal referrals from primary care and community services to the Healthy Babies, Healthy Children (HBHC) Program administered by Hamilton Public Health Services. The HBHC program is a maternal child health program mandated by the provincial government providing home visiting to mothers at risk of poor pregnancy outcomes during pregnancy. In addition, under the HBHC program all new mothers are assessed during the postpartum period and families identified as at risk are eligible to participate in a long-term, mixed model (paraprofessional and PHN) HBHC home visiting program. Eligibility criteria for the pilot study included: 1) being a first-time mother; 2) referral into the program before the end of the 28th week of gestation; 3) ≤ 21 years of age; and 4) assessed to be low-income based on low-income cut-off tables [24], receipt of provincial social assistance, or a self-report of having no income.

We used a single case study approach to move beyond the feasibility study to examine whether the NFP intervention is acceptable to a broad range of stakeholders within the current health care system in Ontario, Canada [26]. In doing so we included an examination of the mothers’ experiences in the NFP program feasibility study. Case study involves exploration, description and explanation of phenomenon within the real life context bounded by time and place [26]. This method is particularly useful when researchers are not in control of variables and when boundaries between the phenomenon being studied and the context of the phenomenon are not easily differentiated [26]. This study was approved by the Hamilton Health Sciences /McMaster University Research Ethics Board.

In order to develop a comprehensive understanding of the mothers’ experiences of participating in the home visiting program, a purposeful sub-sample of women were recruited from among the 108 women in the pilot study. Maximum variation sampling was used to recruit a diverse group of pregnant women and mothers with infants ≤ 12 months, and who were single or partnered, ranged in age from 16 to 21 and at various time points receiving the NFP curriculum. It was estimated that a sample of 15 to 20 women would be needed to reach saturation. The research assistant collecting data for the pilot study approached a purposeful sample of participants in the NFP pilot study, explained the purpose of this portion of the study and obtained written informed consent to participate. Women who agreed to participate received a telephone call to arrange a time and place for an interview. In-depth, semi-structured face-to-face interviews were conducted to explore the women’s perceptions about the NFP program and their experiences within the program. At the end of the interviews, women were asked for permission to be contacted for a follow-up interview within four months to explore their ongoing experiences with the NFP program. One of the researchers (CKL, experienced in maternal infant nursing) and two trained research assistants carried out the in-depth interviews. The in-depth interviews were undertaken at a location convenient for the mothers; usually in the participant’s home. Both the first and follow-up interviews were guided by a semi-structured interview schedule. Examples of interview questions included: What has life been like since you got pregnant/had the baby? What has it been like having a NFP nurse visiting you? And What have you liked/disliked about the NFP program? The interviews lasted 30 to 60 minutes. The participants received a $25.00 gift card to thank them for their participation in the interviews. With the permission of the mothers, the in-depth interviews were digitally recorded. Participants completed a short demographic questionnaire. Observations and a description of the interview context were documented in field notes maintained by the interviewers. An audit trail of decisions made regarding methods, sampling, data collection and analysis procedures was also maintained to support confirmability of the findings.

Data collection and initial analysis were undertaken concurrently. The interviews were transcribed verbatim and each transcript was compared to the recording for accuracy. Conventional content analysis was used to analyze all interviews and field notes [27,28]. Transcripts were read to achieve immersion and to get a sense of the women’s narratives as a whole [28]. The data were reread word by word with a focus specifically on women’s perspectives of, and experiences with, the NFP program to derive codes in order to capture key thoughts and concepts [28,29]. While coding, memos were written regarding first impressions and thoughts emerging from the data. Related codes were then collapsed into broader categories. These emergent categories were used to organize and group codes into themes and subthemes [30]. The researcher CKL coded all of the interviews. To promoted consistency of the emerging themes, double coding was done with the first ten interviews (by CKL and a fourth year nursing student who also was a licensed practical nurse).

## Results

Initially 22 mothers participating in the NFP feasibility study were approached and agreed to participate in the acceptability study. However only 18 of these mothers were subsequently located and completed the first interview between February 2009 and January, 2010. While all of these mothers consented to complete the second interview, only seven of the original 18 mothers were interviewed with four refusing because they were too busy; two mothers no longer had custody of their infants and did not want to be interviewed, one moved and four were lost to follow-up at the time of the second interview. However of the 18 women who participated in the initial interview 10 completed the NFP program. Of the participants who did not complete the NFP program, one moved, two lost custody of their infants and the remaining 5 left the NFP program after their infants turned one year of age. At the time of the initial interview six of the women were pregnant and the remaining 12 had infants < 12 months of age. The average age of the infants at the time of the first interview was four months. Thirteen of the women identified that they were single, and the remaining five were in common-law relationships. All but one of the women reported a total annual income less than $24,000 and 13 of the women identified their main source of income to be from social assistance or another government program. On average the participants had completed a grade 11 education.

The mothers participating in the NFP program were very positive about their experiences in the program. Much of their discussion focused on their experiences with their NFP nurses. Three overarching themes, with subthemes, emerged describing the women’s experiences with and perceptions of the NFP nurses and program. These include: 1. *Getting into the NFP program*; 2. *The NFP nurse is an expert, but also like a friend providing support;* and 3. *Participating in the NFP program is making me a better parent*. Although the themes are thickly interwoven, we will present each of them separately for ease of discussion.

### Getting into the NFP program

Many of the women explained that they were offered the NFP program because they were young, needed support and as first-time mothers, wanted information about pregnancy and parenting. From maternal descriptions of the referral process, it was evident that a wide range of community services were aware of and referring to the NFP program, including: physician or midwifery services, community-based prenatal classes, local Children’s Aid Societies, and their social workers. One woman explained why her doctor referred her to the program, “She just thought it was a good thing for any …young mother …to have the right support and [to answer] any questions I had that she [her doctor] could not answer.” Another woman stated, “I was a patient of the Maternity Centre and the social worker there set me up with the Nurse-Family Partnership program.”

Many of the women shared that they accepted the program because they felt they needed support and had a lot to learn as this was their first pregnancy. One mother explained:

Knowing that there is always someone that you can call if there is anything wrong, and I have like a thousand questions about being pregnant and it’s good to have someone that will be there to answer them.

Another mother stated she accepted the program because she needed support as her boyfriend had left her. She explained that she entered the program, “because I was clearly struggling with the pregnancy thing and being alone in the pregnancy and had asked for support.”

A small number of women indicated that they accepted the program because it was “something to do” while waiting to have the baby. Several were initially ambivalent about participating in the program because of previous experience with other community programs that they felt were “useless.” One mother stated, “I was honestly a little sceptical about it at first…I did not think it would really help, but it’s actually been a really big help.” Another mother shared, “ I thought, ‘Oh it’s just going to be somebody coming to talk to you, check up on you, not really help you.’ I thought it was going to be kind of intrusive at first. But I really enjoyed it”.

### The NFP nurse is an expert, but also like a friend, providing support

In discussion with the mothers about their experiences in the NFP program most mothers focused primarily on the positive relationships they had developed with their NFP nurses. The positive relationships described by the mothers had multiple dimensions which are captured under the following six subthemes: (1) the nurse’s personality; (2) The NFP nurse is “like a friend” who supports you; (3) the NFP nurse is respectful and trusting; (4) the NFP nurse is empowering and an advocate; (5) the NFP nurse is an honest expert; and (6) the NFP nurse is easy to access when you need her help.

#### The nurse’s personality

Most of the mothers when describing their NFP nurses identified that they had many positive personality attributes that facilitated their ability to engage with and develop a working relationship with the nurse. The personality attributes highly valued by the mothers included: a good sense of humour, being friendly, honest, non-judgmental and reliable, coming to the visit in a good mood and having an “easy going” nature. Many of the mothers also emphasized that as the therapeutic relationship with the nurse home visitor became established, they understood and appreciated how much the nurse truly cared about them and their child.

Within this purposeful sample, only a single mother held a negative perspective about the characteristics of her nurse home visitor in commenting that she was “outdated” and further explaining, “She’s a nice lady….fairly easy going most of the time …she’s got the old way of doing things that she likes to go by.” Her viewpoint appeared rooted in contradictory information about infant feeding from her physician and NFP nurse. Despite this opinion, this mother planned to continue to have the nurse visit until her son turned two.

#### The NFP nurse is “like a friend” who supports you

The NFP nurse for many participants was someone they could consistently rely on to provide emotional and informational support. Many of the mothers shared that they experienced precarious social support and relationships with family, friends and their baby’s fathers throughout their pregnancy and beyond. Repeatedly we heard examples of mothers feeling isolated or abandoned during pregnancy by the very individuals they were counting on for social support. One mother explained, “He [the baby’s father] wanted me to get pregnant and then found out I wasn’t having a boy, I was having a girl, and he didn’t want to be there anymore.”

Another mother shared “I lost a lot of my friends during my pregnancy”. Several of the young mothers also described growing up in less than stable environments and as a consequence lacked the support of extended family members. One pregnant woman who had lived in foster care during her childhood because of exposure to maltreatment experienced great sadness that, during this pregnancy and then into the postpartum period, she would not have any support from her mother who ”isn’t as stable as she should have been”.

Many of the mothers identified the NFP nurse home visitor as providing a primary source of social support, particularly emotional support. Several of the mothers used the word “friend” to describe how they perceived the nurse. In her narrative, a young mother shared:

I said, ‘make yourself at home’ and she did. …It didn’t seem like I was sitting there with a nurse, it felt like I was just sitting there with like a friend and so she made me really comfortable and like I could talk to her about anything.

The mothers perceived the nurses as friends because the nurses were easy to talk to and the nurses focused on listening and learning about the mother’s life. The mothers explained that the work of home visiting did not seem “like a job” for the nurses and they perceived that the nurses equally enjoyed the home visiting process. One mother emphasized that she and her nurse, “talked more about my life than I would with other professionals and she knows a bit more about me, so I feel comfortable talking to her about most things.”

Mothers confirmed that they were able to further trust the nurse and open up during the home visits when the nurses were willing to share some of their own personal experiences related to such topics as parenting or healthy relationships. The nature of the conversations during the home visits sometimes included some level of personal sharing and one mother explained, “We talk about life. You know what I mean? We share stories. I know a bit about her husband. She knows a bit about my boyfriend.” As a result, the relationship that developed with the NFP nurse became a highly valued outcome related to program involvement for many of the mothers. One mother shared, *“*The relationship is a bonus because when I joined the program I wasn’t expecting to actually make a relationship with the nurse you know.” Another mother who had problems with addiction and depression, and who had temporarily lost custody of her baby continued to see her nurse on a regular basis. She lacked a support system in Hamilton and had developed good rapport with her nurse who she was continuing to work with her to deal with her issues. She simply expressed, “I feel kind of close to her.” In comparison to other professionals that the mothers had worked with, they perceived that the NFP nurses were consistently more accessible and that they could call and ask for advice and support when they were struggling with issues, not only regarding their pregnancy and baby, but also personal issues such as relationship problems.

A small number of the mothers also disclosed that to them, their nurse idealized their expectations of what a supportive family member, especially their mothers, should provide to them. One mother who had lived in multiple foster homes throughout her childhood and had very minimal contact with her mother, described her relationship with her nurse like the one she thought mothers and daughters should have when the daughter has a baby.

Because of everything that she’s [NFP nurse] *taught* me. I wouldn’t, I wouldn’t have known. Like I don’t have … my mother …[I don’t have contact with her] So like all the stuff I would have called *her [my mother]* up to ask her, I get from my nurse.

All the mothers expressed the importance of having the same nurse visit them throughout their time in the program. They perceived that the quality of their relationship with their nurse would have been threatened had they been visited by multiple nurses. One woman commented:

*[Having the same nurse every visit is important]* because they know you. They know how you are…It’s not a different person *every day* so you have to make that first initial impression *every* single time. It’s *very* important that it’s the same person.

#### The NFP nurse is respectful and trusting

Several mothers liked the feeling that their nurses trusted them. This allowed the mothers, in turn, to trust their nurses. For these mothers being trusted was empowering and very important in the relationship. The mothers’ trust in their nurses is a measure of their level of engagement in the therapeutic relationship as many discussed having difficulty trusting people. A mother shared:

She trusts me and I trust her. It’s just like that. It’s really hard for me to trust people sometimes because I’ve been put down before with friends… but I trust her [NFP nurse] a lot… [I can talk to her] yes about anything.

The importance of being trusted by the nurse was made very clear by a mother who was *“insulted by*” some of the questions about drugs and alcohol in the NFP program questionnaires. She felt that because of the relationship she had developed with her nurse, the nurse should have trusted her not to do drugs because her nurse knows that was *“ not who I am”.* At the same time the mother was comfortable enough to discuss this issue with her nurse and accepted the nurse’s explanation that the questions were a standard part of the curriculum. She felt that it was the nurse’s personality that kept her participating in the NFP program.

#### The NFP nurse is empowering and an advocate

Several mothers spoke about how the nurses empowered them. They felt they were in control of the activities they did with their nurses. They liked the fact that they set the learning agendas with their nurses based on their own needs instead of the nurses deciding for them.

She asks me what I want her to bring in [to the home visit] so that’s kind of nice. She suggests things she could bring….and asks me if I want to see any of that?…she says she will bring it if I want her too.

In addition mothers were pleasantly surprised that they had control over scheduling home visits. They shared: “They are willing to go around your schedule and set up days that are good for you and not just like convenient for them.” Most important, the mothers in this study appreciated that the structure of the program allowed for their nurses to be accessible and flexible in changing or scheduling new home visit appointments.

Many of the mothers discussed how good it felt when their nurses gave them positive reinforcement related to their self-care and parenting. As one mother said: “She’s very encouraging and sometimes just hearing that you’re doing a good job makes the biggest difference in the world, especially with being such a young parent*.”* Another mother stated: “She makes me feel really good. She doesn’t make me feel like I did anything wrong, like I raised her wrong. She kind of makes it like it’s not your fault, it’s your first time, you’re learning.”

Some of the women shared that their nurses advocated for them when dealing with community agencies. One mother who was being monitored by the Children’s Aid Society (CAS) discussed how her nurse helped her deal with CAS and supported her when the agency child welfare worker visited. “The NFP [nurse] gives me good tips on stuff like how to deal with them [CAS]…. [my NFP nurse] comes to my meetings [with CAS] too…. [that] shows CAS like [I] do what I need to do with my son.”

*The NFP nurse is an accessible honest expert who gives advice and answers questions.* The mothers valued the fact the nurses were knowledgeable health care professionals. They described the nurses as credible and honest experts they could rely on. Most of the mothers felt they could ask the nurses about anything. Furthermore, the information provided by the nurses was perceived as helpful. The mothers expressed that the nurses were skilled in assessing their information needs and providing education they could understand.

For the new mothers, they particularly valued the nurse’s expert opinion about their infant’s health and development. Nurses were also recognized for their skills in conducting physical assessments of the mothers’ infants. Many of the mothers found the information provided by the nurses comforting, as the nurses were able to normalize and explain new situations the mothers were experiencing. As one mother explained:

And then every time I had to ask her a question about anything like when his umbilical started to fall off, I needed her to *check it* to make sure it looked right because I didn’t think it did but it was perfectly fine. So that was good. She’s really, she’s really cool.

For many mothers knowing they had easy access to expert information from their nurse was reassuring. One mother shared:

Knowing that there’s always someone that you can call if there’s anything wrong, and I have like a thousand questions about being pregnant and it’s good to have someone that will be there to answer them. Because I ask everyone and they obviously don’t know accurate answers, they just tell me what they think. But a public health nurse they know the actual answer, so I like to know.

Although the mothers saw their nurses as professionals, the mutual respect and trust that had become part of their relationships empowered them to see themselves on the same level as their nurses, i.e. person to person. This allowed them to ask their nurses health questions they did not feel comfortable asking other health care professionals. One mother stated:

She’s more to me a person on the street that I can go and meet, not just like a professional. I know she’s a professional but it’s just easier on me knowing that she’s not I’d say uptight as what a doctor would be or something.

#### The NFP nurse is easy to access when you need her help

Most of the mothers did not have access to cars, nor could they afford regular use of taxis. Use of public transportation became more difficult once their infants were born. The mothers felt that the home visits made it much easier for them to engage with their NFP nurses on a regular basis. One mother stated, “That she comes to me so I don’t have to try and get out my door and go see her like at an office that I’d probably have to take a bus to…because [public] *transportation* is really difficult…with a baby.”

Access to the nurses by telephone was also very important to the mothers. All the mothers shared that they could contact their nurses by telephone if they had questions or needed their help. In addition they knew that they could rely on the nurses getting back to them in a timely manner when they called them. One mother said:

She’ll answer her phone or she’ll call me back. After leaving a message she’ll call me back right away….[She will call back] sometimes same day, sometimes the next morning depending on what time it is throughout the day.

The quick access to their nurses by telephone was extremely important to many of the women if they were worried about themselves or their infants. As one mother shared:

It’s a lot of *stress* off my chest [if I can call my nurse] because sometimes I think that something’s *wrong* when it’s *not*. Sometimes I’m just *panicking* because it’s the first time and never had this happen before, right? So I’m just learning.

Several mothers felt that after their infants were born the home visits with their NFP nurses sometimes became too long. They explained that since their baby was born they were much busier than when they were pregnant. Many had returned to school and found attending school and looking after their infants overwhelming and exhausting. The home visits were one more activity to fit into their hectic schedules. This is reflected in the following comment:

I know that the visits were getting like very long but that was like the only thing, like we’d talk for so long. That was the only thing that the visits got like up to like maybe an hour and a half, 2 hours at a time….We kind of cut it down probably like a half an hour every time. She [nurse] would probably have liked an hour a visit.

### Participating in the NFP program is making me a better parent

Most of the mothers shared the view that by participating in the NFP program they were becoming better parents. They identified that they had learned a lot from their nurses regarding self care, healthy living during pregnancy, breastfeeding, interpreting infant behaviour, infant developmental milestones, parenting, basic physical care of their infants, healthy relationships with partners and family members and available community resources. They shared that with each visit from their nurses they were learning new “stuff” that they wanted to know to make them good mothers. For example women interviewed during pregnancy shared that they learned important information to help them have a healthy baby. A mother commented:

Like she brings in things [videos on birth, breast feeding, information on good nutrition] and … like what to look out for when you’re pregnant and after… I never knew half of those things. So she’s already helped me a lot.

In the early weeks after their babies were born mothers shared that their nurses not only helped them learn the skills needed to physically care for their babies, but also helped them understand their babies’ behaviour and how to relate to their babies. One mother whose baby was 3 weeks old explained:

Now she’s [NFP nurse] starting to teach me some lessons. Yesterday we did a thing on how a baby learns about *love* through *trust and security*. I thought that was cool. I never knew a baby could like *take in* love like *we* can because they don’t really know. But I thought that was pretty cool that they learn to trust you [when you respond to their needs]… that’s how they learn how to love.

Another mother when asked what she felt she was getting from participating in the program stated:

It’s made me learn a lot more to make myself a better mother than if I didn’t know anything at all and I’m just winging it …taking care of him.…It’s going to probably help me learn to make sure that as he’s growing up. I’m going to teach him different things…the proper way. He’s learning exactly what he’s supposed to be by this age and all that.

Several women in the study who had partners felt that participation in the NFP program was helping them and their partners become good parents. One mother explained:

I think he [partner] likes it because in a way it’s helping us build up our skills for parenting as well as build up our skills for making sure the baby’s healthy and ourselves are healthy…I think …she’ll [NFP nurse] help me because she’ll help me raise a healthy family and she talks about like healthy relationships with your family and with your boyfriend and everything. So it’s not just about like me and the baby, it’s about the whole thing.

## Discussion

Knowledge of mothers’ experiences participating in the NFP program is extremely important for the development of a comprehensive understanding of critical aspects of the program that make it effective in improving health and social outcomes for mothers, their children and families in general. This study is one of the first to examine the NFP program from the perspective of clients participating in the program. Our findings contribute valuable new insights and start to address the paucity of information about the therapeutic relationships between ‘at risk’ mothers and NFP nurses that have been hypothesized to play a key role in the effectiveness of the NFP program [13]. Overall, the findings suggest that the mothers’ experiences in the NFP program were very positive and highlight the critical importance of the nurse-client relationship. Several of our findings are similar to those reported in previous studies examining mothers’ perspectives on home visiting in general such as the importance mothers placed on feeling respected [12,15,21,31] and not patronized or judged by their home visitors [32], appreciating the support they received from [12,15,23,31]the home visitor [14,15,33] and the flexibility of program delivery [31,33]. In addition some of our findings are similar to those reported in previous studies specifically exploring mothers’ experiences with nurse home visiting. For example mothers valued home visiting nurses who did not lecture or tell them what to do or how to raise their children [12,21]; with whom they felt connected and trusted [12]; and who provided them with positive reinforcement regarding their parenting skills [21]. As in previous research on parents’ concerns about allowing PHNs into their homes, a few women in this study were initially worried about entering the NFP program for fear the nurses would be ‘agents of the state’ and serve a surveillance function [12,34,35]. However none of the women expressed ongoing concern once they had developed a relationship with their nurses. Consistent with the NFP nurses’ strengths based focus, it is likely that the nurses practiced a “caring strategy of watchful waiting” and “expectant management” rather than that of surveillance which has been postulated to undermine the development of strong nurse-client therapeutic relationships (p.416) [34].

Many of the women in our study were surprised by the positive relationships they developed with their nurses. Some even considered their nurses to be ‘friends’ they could confide in and trust. (‘Friend’ in this case is likely the lay term for therapeutic relationship as the women never appeared to lose sight of their nurses’ professional role in their relationship.) Our findings support the theory postulated by Falk Rafael that good therapeutic relationships are developed between home visiting nurses and mothers when the nurses are non-judgemental and help mothers to become empowered to increase control over their lives and health [19]. However our findings provide more insight into specific factors that are important to the women in their relationships with nurses. The women in this study valued the feeling that the nurses cared about them and that their relationship was not just ‘a job’ for the nurses. The women felt the nurses were engaged with and interested in them as individuals, helped and supported them with what they needed and made them feel good about their successes in parenting. As one mother explained, she had a ‘person to person’ relationship with her nurse even though her nurse was a professional. The non-hierarchical and empowering relationships that many of the women perceived are considered by nurse experts to be the most therapeutic [35]. This type of relationship allows nurses to engage more fully with their clients, enables mutual exploration of the clients’ everyday lives and allows collaboration to more comprehensively address struggles, needs and concerns [36,37]. The approach is consistent with, if not imperative to, the effective implementation of the NFP’s strengths based visit guidelines in which the nurse recognizes that the mother is the expert in her own life and helps her identify her desired goals. The nurse together with the mother focuses on the mothers’ strengths and the resources needed to move toward these goals, and on solutions to issues, small steps and provision of feedback [2]. Our findings indicate that therapeutic relationships that have an imbalance of power would not be effective in delivering the NFP program. This type of therapeutic relationship is more likely to be authoritarian and unfairly partial [38]. Nurse client relationships with an imbalance of power often result in nurses providing routine disengaged practices with little effectiveness as the practices are not tailored to the needs of clients [13,39,40].

For many young, low income, pregnant women a major motivator to participate in nurse home visiting programs may be a lack of adequate social support. Participants in our study shared that they entered the NFP program in order to obtain needed support. Consistent with the findings of previous studies on low-income mothers, most of the participants in our study experienced precarious social support [22,41-43]. Their NFP nurses helped to fill this gap as mothers reported that they received both emotional and informational support during home visits.

As such, the support provided by NFP nurses may be a key critical factor, foundational to the effectiveness of the NFP program, enabling young mothers to engage in activities that will move them forward to employ healthy parenting and healthy self- development. Young, expectant and/or parenting, low income mothers often struggle with the responsibility of motherhood, material deprivation, and are at high risk for psychological distress [44-47]. Low social support and poverty combined with associated stressors place children at greater risk for child maltreatment, unintentional accidents and other negative health outcomes [48-50]. Adequate social support decreases the impact of stressful life circumstances on health outcomes [48,51,52] and aids in healthy childbirth transition [53,54]. The support received by the mothers from their NFP nurses likely provided a buffer to the daily stressors. Mothers who receive appropriate social support have: 1) more resources to meet their children’s needs [55]; 2) lower incidences of postpartum depression [56,57]; 3) fewer problems with maternal alcohol and drug abuse [58]; 4) an increased rate of maternal education completion [59]; 5) fewer unintentional infant injuries [60]; and 6) lower rates of child abuse [61].

All the women interviewed felt their participation in the program was helping them to become good mothers. They identified they had learning needs about pregnancy, infant care and parenting before entering the program. This was one of the reasons they gave for agreeing to participate in the program. Similar to findings in previous research [62], mothers in this study valued receiving sound advice and correct information from their nurses. From the participants stories it became apparent that the NFP curriculum was meeting their learning needs. The strong positive relationship they had developed with their nurses, recognition of their nurses as experts (with the exception of one mother who had received information from her physician that contradicted the information from her nurse) and the accessibility of their nurses was key to empowering them to identify their learning needs and to ask questions. This included questions they were not comfortable asking other health care and social service professionals. Interestingly some of the mothers found that the nurses were not only assisting them to be good mothers but were also helping their partners become good fathers.

Our exploration of mothers’ perceptions of and experiences in the NFP program provides valuable insights into some of the factors that make the NFP program acceptable to mothers and effective in improving maternal and child health and social outcomes. As reported earlier, several of our findings have been reported in previous studies of mothers’ experiences in other nurse and non nurse led home visiting programs yet many of these programs do not have demonstrated effectiveness [12,14,23,31-36]. The effectiveness of the NFP program can likely not be linked to individual aspects of the program such as the therapeutic relationships the nurses establish with mothers. Rather the effectiveness of the program is likely due to the synthesis of all the components of the program. This includes the motivation of the women to become good mothers and wanting to learn more about pregnancy and parenting; the expert nurses and the extensive NFP training and professional support they receive; the continuity of provider; the importance of the nurses’ professional expertise to the mothers; the theoretical underpinnings of the program, i.e. human ecology, self-efficacy, and human attachment; the quality of the nurse client therapeutic relationship; the nurses’ strengths based approach; a curriculum that is relevant to ‘at risk’ mothers; the ease of access to the NFP program; the intensity of the visits; and the prolonged engagement. If the assertion that the NFP program’s effectiveness is dependent on the synthesis of all the program components is correct, the development of new home visiting programs for at risk mothers and children may be ineffective if only a few components of the NFP are implemented. Further research is required to continue to tease out the underlying processes that make the NFP an effective intervention. This knowledge is needed to continue to improve the effectiveness of the NFP program, to effectively adapt and deliver the program in new contexts and to help inform the development of new home visiting programs.

### Limitations and strengths

This study has both strengths and limitations. Our study focused on the experiences of mothers in the NFP program from the time of entry into the program until the child’s first birthday. More insights into the experiences of mothers in the NFP program may emerge if we interview mothers within the second year of their child’s life and after completion of the NFP program. Although our second interview with participants indicated that they continued to have positive experiences within the NFP program we were only able to re-interview 7 of the 18 participants. It is possible that women who did not participate in the second interview were unhappy with the program; however this may not be the case. Three participants had exited the NFP program at the time of the second interview for the following reasons; one had moved out of the region and two had lost custody of their infants and no longer wanted to participate. Five of the participants who did not partake in the second interview graduated from the NFP program when their children turned 2 years of age, and the remaining 3 participants who did not participate in the second interview exited the program after their infants turned one year of age (after the time of the second interview). Our difficulty locating some of the participants for the second interview was likely due to factors related to poverty in this population which can lead to multiple household moves and an inability at times to afford a telephone. These factors make it challenging for researchers to follow-up with participants [22]. Another possible explanation for our inability to re-interview some of the participants may be related to interviewee fatigue as the participants were also completing multiple interviews for the NFP feasibility study. Similar to other studies examining mothers’ experiences with home visiting, participants in our study had few criticisms of and were very positive about the NFP program [15,33]. This may be due to their overall satisfaction with the program and therefore they did not want to complain about aspects of the program they found dissatisfying. It may also relate to their limited experiences with home visiting services and thus they had nothing to compare to the NFP program. Strengths of the study include the qualitative design which allowed the mothers’ perspectives to emerge through the in-depth interviews and qualitative analysis. This approach allowed us to develop a good understanding of the mothers’ experiences participating in the NFP program and to start to understand the processes at work in the program that are effective in improving both maternal and child outcomes.

## Conclusions

Examining women’s experiences in the NFP program is an important step in uncovering the ‘active ingredients’ in effective home visiting. To date the NFP program is one of only a few home visiting programs to demonstrate effectiveness in improving health and social outcomes in both vulnerable mothers and their children [17,18]. Our exploration of the mothers’ experiences within the NFP program provides important new information from the mothers’ perspectives about why they entered and engaged in the NFP program, how they felt about what they were learning, their NFP nurses and the home visits, and what they thought participating in the program was doing for them and their child.

As our study was limited to the examination of the mothers’ experiences until their child’s first birthday, further research is required to explore the experiences of women in the second year of the NFP program. Retention of families at risk in home visiting programs is often very challenging [61]. Given our finding that the participants were generally very positive about the NFP program and planned to continue until graduation, we developed limited insight into why some women leave the NFP program before completion. Future research should examine the experiences of women who exit the NFP program prematurely to help inform the development of strategies to prevent unnecessary program attrition.

This study should be replicated in other locations to develop a comprehensive understanding of the spectrum of mothers’ experiences in the program across multiple sites and different contexts (for example in urban versus rural communities, and jurisdictions with publically funded versus privately funded health care). Finally research is needed to demonstrate the effectiveness of the NFP program in meeting the multifaceted needs of at risk mothers and their children within the Canadian context.

## Abbreviations

NFP: Nurse-Family Partnership; HBHC: Healthy Babies Healthy Children; PHN: Public Health Nurse.

## Competing interests

The authors declare that they have no competing interests.

## Authors␁ contributions

CKL conceived of and designed the mothers’ experiences component of the larger study, collected, analyzed and interpreted the data and drafted the manuscript. SMJ conceived of and participated in the design of this study, data interpretation, and revisions to the design of the study and revisions to the manuscript. HM conceived of and designed the larger NFP pilot study and contributed to revisions to this manuscript. All authors read and approved the final manuscript.

## Pre-publication history

The pre-publication history for this paper can be accessed here:

http://www.biomedcentral.com/1472-6955/11/15/prepub
